# The Feat of Packaging Eight Unique Genome Segments

**DOI:** 10.3390/v8060165

**Published:** 2016-06-17

**Authors:** Sebastian Giese, Hardin Bolte, Martin Schwemmle

**Affiliations:** 1 Institute of Virology, University Medical Center Freiburg, Hermann-Herder-Strasse 11, 79104 Freiburg, Germany; sebastian.giese@uniklinik-freiburg.de (S.G.); hardin.bolte@uniklinik-freiburg.de (H.B.); 2 Spemann Graduate School of Biology and Medicine, University of Freiburg, 79104 Freiburg, Germany

**Keywords:** influenza virus, vRNP, genome packaging, formation of genome sub-bundles, packaging sequences

## Abstract

Influenza A viruses (IAVs) harbor a segmented RNA genome that is organized into eight distinct viral ribonucleoprotein (vRNP) complexes. Although a segmented genome may be a major advantage to adapt to new host environments, it comes at the cost of a highly sophisticated genome packaging mechanism. Newly synthesized vRNPs conquer the cellular endosomal recycling machinery to access the viral budding site at the plasma membrane. Genome packaging sequences unique to each RNA genome segment are thought to be key determinants ensuring the assembly and incorporation of eight distinct vRNPs into progeny viral particles. Recent studies using advanced fluorescence microscopy techniques suggest the formation of vRNP sub-bundles (comprising less than eight vRNPs) during their transport on recycling endosomes. The formation of such sub-bundles might be required for efficient packaging of a bundle of eight different genomes segments at the budding site, further highlighting the complexity of IAV genome packaging.

## 1. Introduction

The viral genome of Influenza A viruses (IAVs) is composed of eight negative-sense viral RNA (vRNA) segments, complexed with viral proteins to form ribonucleoprotein (vRNP) complexes [1,2,3]. The 3′ and 5′ termini of each vRNA are bound by the polymerase complex, while the remaining molecule is encapsidated by multiple copies of the viral nucleoprotein (NP) [2,3,4]. Typical of IAV is the ability to exchange (reassort) viral genome segments in cells that have been co-infected with different IAV [5,6]. This feature increases successful adaptation to new hosts (e.g., by escaping immune pressure) and has often preceded the emergence of pandemic IAV strains in the past [5]. Although a segmented genome may be a major advantage to overcome restrictions in novel hosts, it demands a highly sophisticated mechanism that coordinates assembly and incorporation of a complete vRNP set consisting of the eight distinct genome segments into viral particles (reviewed in [7]).

It is widely established that genome-packaging sequences residing mainly in the 3′ and 5′ termini of each vRNA [8,9] ensure the formation of a complex of eight distinct vRNPs and their incorporation into budding particles, a process that we define as ‘genome packaging’. Intriguingly, there is an emerging concept that the association of two or more distinct vRNPs into so called vRNP sub-bundles, visualized by advanced fluorescence *in situ* hybridization (FISH) techniques [10,11], already occurs *en route* from the nucleus to the actual budding site, a process we refer to as ‘genome bundling’.

This review summarizes the current knowledge regarding late steps during IAV infection and discusses whether ‘genome bundling’ and ‘genome packaging’ are interdependent events or not.

## 2. Nuclear Export of vRNPs

Upon acidification of the endosome and consequential membrane fusion, vRNPs are released into the cytoplasm and actively translocate to the host cell nucleus via importin alpha/beta [12,13,14]. Contrary to most RNA viruses, IAV rely on the host nuclear transcription machinery to initiate vRNA replication and transcription (reviewed in [15]). In a process called ‘cap-snatching’, the polymerase basic protein 2 (PB2) subunit of the viral polymerase binds to the cap structure of nascent cellular pre-mRNAs, leading to their subsequent cleavage of 10-13 nucleotides downstream of the 5′ end by the polymerase acidic protein (PA) subunit [16,17,18]. These short mRNA fragments serve as a primer for viral mRNA synthesis, which is carried out by the subunit polymerase basic protein 1 (PB1) [19,20,21,22]. Subsequently, newly translated polymerase subunits and NP enter the nucleus and allow amplification of vRNPs via complementary ribonucleoprotein (cRNP) complex intermediates [23,24,25].

Newly synthesized vRNPs are then exported from the nucleus, a process mediated by a multiprotein complex including cellular chromosome region maintenance 1 (CRM1), the viral nuclear export protein (NEP) and viral matrix protein 1 (M1) [26,27,28,29,30,31,32]. The so-called daisy-chain model, initially proposed by Akarsu and colleagues and later refined by Brunotte and colleagues suggests that nuclear vRNPs associate with NEP and M1 in order to be linked to the cellular export receptor CRM1, thus promoting their transit through nuclear pores in a GTP-binding nuclear protein Ran (Ran^GTP^)-dependent manner [26,28]. The activities of such multiprotein complexes are often coordinated by post-translational modifications (PTMs). Accordingly, it has been demonstrated that nuclear export relies not only on the vRNP-NEP-M1 protein complex formation itself, but also requires SUMOylation of M1 [33] as well as phosphorylation of NP [34] and to a minor extent NEP [35]. A recent study further identified the cellular human immunodeficiency virus (HIV) Rev-binding protein (RBP) as a critical factor during vRNP export. The authors speculate that RBP might interrupt CRM1-Ran^GTP^ binding through GTP hydrolysis in the cytoplasm and hence mediate vRNP release from this nuclear-export-complex [36]. Moreover, there is evidence that IAV infection triggers Caspase-3 activation [37], which eventually results in nuclear pore destruction at later time points during infection [38]. This suggests that in addition to the above-mentioned CRM1-Ran^GTP^-dependent nuclear vRNP export, another pathway could be functional during the ongoing infection.

While the vRNP nuclear export mechanism itself has been extensively studied, controversy remains as to whether newly synthesized vRNPs are exported from the nucleus individually [11,39] or as vRNP bundles [10]. By means of FISH analyses, Chou and colleagues studied the co-localization of two viral segments during the course of infection in MDCK cells. Remarkably, they could reveal that the viral PB2 segment only minimally co-localizes with any of the other investigated viral genome segments (PB1, PA, NA, NP, M, NS) at 4 h post-infection (hpi), when nuclear vRNP export presumably takes place, suggesting that vRNPs are individually translocated to the cytoplasm [11]. These findings are further corroborated by a recent fluorescence correlation spectroscopy (FCS) analysis providing no evidence for the presence of multi-vRNP complexes within the nucleus [39]. In sharp contrast, using a four color FISH assay, Lakdawala and colleagues observed the co-localization of vRNP bundles containing the viral PB2, PB1, PA and NP segments in close proximity of the nuclear membrane at 8 hpi (again using MDCK cells) [10]. The discrepancies of these findings remain unclear but may be related to the use of different IAV strains, technical differences and/or the different time points post-infection with which the co-localization studies were performed. Mentionable, although FISH is a powerful method in detecting genome segments of IAV, a general limitation of this method is the rather low resolution of approximately ~240 nm [10], which depends on multiple factors such as the microscope itself, the objective and sample preparation. As a result, physical interaction between two vRNPs cannot be conclusively demonstrated by FISH. For these reasons, alternative approaches, including Förster resonance energy transfer (FRET), are required to demonstrate whether vRNPs are exported from the cell nucleus as individual segments or vRNP bundles (see also Isel et al., this issue).

## 3. Perinuclear vRNP Accumulation at the Microtubule-Organizing Center

Once exported from the nucleus, vRNPs accumulate in the perinuclear region within close proximity to the microtubule-organizing center (MTOC) [11,40,41,42] and the associated endosomal recycling compartment (ERC) [43]. Following accumulation, they are actively transported to the apical plasma membrane (APM) by hitchhiking onto Ras-related in brain (Rab)11^GTP^-bound vesicles [40,44,45,46]. These transport vesicles emerge from the ERC [43], which is characterized by the presence of Rab11 GTPases and members of the Rab11 family of interacting proteins (FIPs) [47].

To facilitate vRNP transport to the plasma membrane, IAV usurps the cellular endosomal recycling system [40,41,44,45,48]. Recent evidence suggests that IAV infection prompts a currently unknown guanine nucleotide exchange factor (GEF) [43] to convert Rab11^GDP^ into the GTP-bound form Rab11^GTP^. This conversion is necessary in order to tag this protein to recycling endosomes [49]. Meanwhile, MTOC maturation is achieved by IAV mediated recruitment of the cellular factor Y-box binding protein-1 (YB-1) [43], a protein which anchors newly synthesized microtubules onto the MTOC [43] to enable the eventual endosome transport. Finally, Rab11^GTP^-positive recycling endosomes localize close to the MTOC [43], where they accommodate vRNPs by directly binding to Rab11^GTP^ [41,45]. It is worth noting that although YB-1 seems to be directly involved in numerous activities at the perinuclear region [50] and YB-1 knockdown consequently resulted in both reduced Rab11 accumulation at the MTOC and impaired viral titers, particle release was only marginally reduced [43]. This observation suggests the presence of additional cellular factors involved in vRNP-loading onto Rab11^GTP^ positive recycling endosomes. Although it can only be speculated, one candidate might be RBP, which was also found to be localized within the perinuclear area where vRNP-loading occurs [36]. Similar to the case of vRNP export from the cell nucleus, it remains undetermined whether single vRNPs or vRNP bundles are loaded onto Rab11^GTP^-positive recycling endosomes [10,11].

## 4. Rab11^GTP^-Mediated vRNP Transport Towards the Apical Plasma Membrane

Once vRNPs are bound to Rab11^GTP^, recycling endosome complexes are actively transported along microtubules [45] accumulating without membrane fusion below the plasma membrane [40].

In uninfected cells, vesicle transport along microtubules and actin filaments is mediated by multiple motor protein complexes consisting of the vesicle membrane-bound Rab11^GTP^, Rab11-FIPs and distinct motor proteins, including dynein, actin motor protein myosin Vb (MyoVb) or the kinesin family member (KIF) 5a [51]. In general, Rab11 GTPases are known to regulate exocytic and recycling processes in the direction of the cell membrane [51,52]. FIPs, which predominantly interact with Rab11^GTP^, function as adaptor proteins between Rab11 and its respective motor protein [44,51].

Intriguingly, several studies revealed an unusual punctuate pattern of cytoplasmic Rab11, indicating aggregate formation upon IAV infection [36,40,53]. This atypical Rab11 distribution pattern suggests that vRNPs do not simply utilize, but modify, the vesicle transport system. Consistently, while overexpression of FIPs lacking Rab binding domains (FIP-∆RBDs) has no effect on vRNP accumulation (and most likely loading on endosomes) within close proximity of the MTOC, overexpression of Rab11-FIP RBD fragments alone impaired vRNP localization to the APM [45]. Vice versa, excess vRNPs seem to directly compete with FIPs for Rab11^GTP^-binding, resulting in vRNP transport along microtubules accompanied by the simultaneous hindrance of cellular transport pathways [53]. Strikingly, while vRNP transport is thought to be conducted by motor proteins [45], no adequate candidate has been identified so far. One plausible candidate might be KIF5b, a kinesin family member which has been shown to be associated with the viral polymerase subunits PB2 and PB1 [54]. In this scenario, the viral polymerase, rather than a FIP, may function as an adapter between Rab11^GTP^ [41,46] and the motor protein KIF5b [54].

As mentioned earlier, due to recent evidence obtained via FISH, it is believed that vRNPs accumulate on Rab11^GTP^ vesicles *en route* to the APM [10,11]. Using a four color FISH assay, genome bundles containing all segments were detected at the APM where packaging occurs. In contrast, vRNP sub-bundles lacking at least one genome segment are found within the cytoplasm in close proximity to the nucleus [10]. Thus it is believed that Rab11^GTP^ vesicles that emanate from the perinuclear region via microtubules carrying such vRNP sub-bundles [10,41,45] finally gather within distance and without fusion to the plasma membrane [40].

Despite intensive research, the answers to several questions regarding Rab11-mediated vRNP transport remain elusive. Aside from the transport mechanism itself, the precise role of genome bundling [10] is poorly understood. Evidence shows that Rab11-positive vesicles serve as platforms that allow vRNP bundle formation [10,11]; however, the molecular determinant inducing bundle formation prior to packaging is unknown. It also remains unclear why vRNP-Rab11 vesicles stall their movement prior to membrane fusion [40]. Finally, the mechanism inducing vRNP release from Rab11 vesicles remains to be determined. Generally, cellular recycling processes rely on Rab11^GTP^ hydrolysis by GTPase activating proteins (GAPs) and subsequent action of Rab GDP-dissociation inhibitors (GDIs) to recycle Rab11 proteins [49]. Hence, it is feasible that, following microtubule release, IAV infection upregulates yet unknown GAPs close to the APM, thereby disrupting vRNP-Rab11 binding.

## 5. Genome Packaging

Newly synthesized vRNPs, together with the remaining viral structural proteins hemagglutinin (HA), neuraminidase (NA) and the viral matrix proteins M1 and M2, are assembled directly at the apical plasma membrane to form progeny virus particles. Virus assembly is spatially organized in the ‘viral budozone’ [55], a large stabilized membrane domain, constituted of several coalesced lipid raft microdomains [56,57]. HA and NA are intrinsically targeted to lipid rafts [58,59,60] during their apical trafficking [61] and define the site of the ‘viral budozone’. In contrast, M2 probably traffics independently of HA and NA to the plasma membrane [62] and primarily localizes to the fringe of the ‘viral budozone’ [57], albeit the molecular properties determining its localization remain elusive [63]. Targeting of M1 to the plasma membrane is presumably achieved by direct interactions with either HA, NA or M2 during their apical transport [64,65,66]. There is a growing body of evidence indicating that the actual presence of a fully assembled genome bundle, consisting of eight unique vRNPs at the budding site, represents the major driving force for the efficient formation and subsequent release of progeny viral particles. Indeed, omission of one particular genome segment during reverse genetic-based virus rescue experiments, in the presence of the cognate viral protein(s), dramatically reduces [67,68,69] or totally abolishes [70] the production of infectious viral particles. Likewise, introducing silent mutations into functionally important regions of the genome packaging sequences, located at the 3′ and 5′ vRNA termini, drastically diminishes the total number of released viral particles as counted by electron microscopy [71,72]. Strikingly, electron microscopy analysis of infected cells revealed no accumulation of viral particles stalled on the cell surface during the pinching-off process, suggesting that in the absence of a packaging-competent complex of eight distinct vRNPs, not only is particle release impeded but so is the preceding step of the viral budding process [71].

Although the precise mechanism of late vRNP trafficking to the ‘viral budozone’ currently remains poorly understood, sophisticated studies using electron microscopic tomography succeeded in capturing the incorporation of the complete vRNP bundle into nascent viral particles [73,74,75,76,77]. During this process, the eight vRNPs of different lengths appear to align perpendicular to the budding tip. Notably, whether packaged vRNPs are oriented in a parallel or antiparallel fashion remains controversial [73,74,75,78]. Irrespective of their orientation, the complex of eight distinct vRNPs associates with the interior of the nascent virion [77]. It was shown that mutations in the cytoplasmic tails of HA, NA and M2 not only alter vRNA incorporation efficiency but also reduce the total numbers of released infectious viral particles [66,79], hence it is believed that HA, NA and M2 are involved in the recruitment of vRNPs to the budding tip. The precise role of M1 during vRNP recruitment is currently unclear, however, due to its reported interactions with all transmembrane proteins and the vRNPs [64,65,66,80,81,82], M1 is believed to function as an adaptor, connecting the vRNPs to the viral membrane during ‘genome packaging’.

During ‘genome packaging’, the eight vRNPs arrange in a marked ‘(7 + 1) order’ with seven peripheral vRNPs surrounding a central one [76,83]. Notably, all studies found the vRNPs to be visually interconnected, suggesting that interactions play a decisive role during the incorporation process. In particular, using 3-dimensional (3D) electron tomography, Marquet’s group observed a platform-like structure underneath the viral matrix layer, probably containing the viral polymerase as well as the postulated terminal genome packaging sequences of all vRNPs [73,74,75]. Kawaoka’s group not only detected direct contacts between vRNPs, but also interconnecting ‘string-like structures’ resembling vRNA. Strikingly, contacts between vRNPs were found to span the whole length of the vRNP bundle [77], suggesting, that interactions between vRNPs may involve other vRNP components or internal vRNA regions apart from the previously described terminal genome packaging sequences. Indeed, Gavazzi and colleagues described internal vRNA regions of two viral segments to be functionally important for ‘genome packaging’ [84].

These findings not only corroborated the involvement of RNA-RNA interactions between different segments in guiding ‘genome packaging’ but also suggested a mechanistic model for this process. Since a portion of the encapsidated vRNA is thought to retain secondary structures [85,86], it has been proposed that small vRNA hairpins (so-called kissing loops) may protrude from vRNPs, thereby facilitating interactions between complementary RNA regions of different viral segments [84].

Altogether, these findings underscore the likelyhood of the previously defined genome packaging sequences to be the key determinants required for the assembly of eight distinct vRNPs and its subsequent incorporation into viral particles.

## 6. Genome Bundling and Genome Packaging: Interconnected or Not?

**Figure 1:**
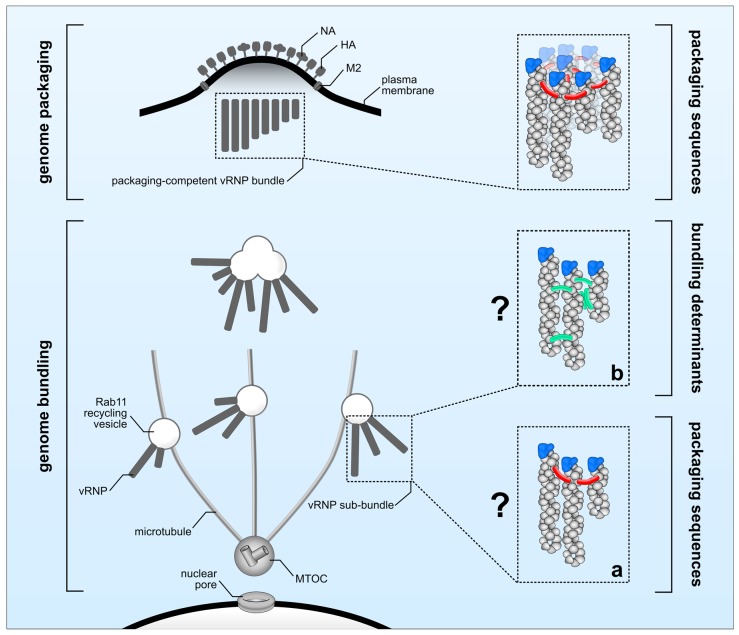
Molecular determinants regulating genome bundling and genome packaging. After accumulation close to the microtubule-organizing center (MTOC), viral ribonucleoprotein (vRNP) complexes are transported as vRNP sub-bundles on Rab11 recycling vesicles (genome bundling) towards the plasma membrane to be incorporated as a complex of eight different vRNPs into budding virions (genome packaging). ‘Genome packaging’ (upper panel) is regulated by packaging sequences (indicated in red). The molecular elements required for ‘genome bundling’ (lower panel) are currently unknown and could involve either (**a**) packaging sequences (red) or (**b**) yet unknown bundling determinants (green) including secondary RNA structures or host factors.

There is a substantial body of evidence indicating that genome packaging sequences localized within the 3′ and 5′ ends of each vRNA are crucial in guiding the incorporation of the eight unique vRNPs into budding viral particles [8]. Here, we defined this process as ‘genome packaging’ (Figure 1, upper panel). However, several open questions regarding the molecular determinants controlling ‘genome bundling’ and the nature of vRNP sub-bundles remain elusive (Figure 1, lower panel). It is unclear whether the observed co-localization of distinct vRNPs *en route* to the plasma membrane reflects physical interactions. Microscopy techniques previously used to investigate ‘genome bundling’ do not provide sufficient resolution to answer this question, leaving the possibility that co-localization simply reflects two vRNPs being in close proximity of one another without physically interacting, an event that is likely to occur when newly synthesized vRNPs accumulate in proximity of the plasma membrane. In support of this scenario, it was recently shown that IAV infection leads to Rab11-positive vesicle clustering giving rise to cytoplasmic vRNP hotspots [53]. Thus, ‘genome bundling’ would be a random process, and no vRNP sub-bundle composition should be significantly favored over another. However, in sharp contrast to this scenario, vRNP sub-bundles are consistently found to be composed of specific segments [10,11], suggesting ‘genome bundling’ to be a non-random process that is able to discriminate between different vRNPs. Albeit conceivable that vRNP components are determining this process, to date direct evidence is still missing as to whether ‘genome bundling’ is indeed mediated by physical vRNP interactions or, for example, cellular factors. Assuming that physical interactions govern the formation of vRNP sub-bundles, the actual nature of these interactions still remains obscure. It is tempting to speculate that the previously defined genome packaging sequences are involved not only in ‘genome packaging’ but also in ‘genome bundling’. Thereby, both processes would be tightly interlinked by the action of the genome packaging sequences guiding the sequential maturation of vRNP sub-bundles *en route* to the plasma membrane into a complex of eight distinct vRNPs to be eventually incorporated into progeny particles at the budding site. Although this would be an appealing model, there is evidence that both processes might occur at least partially independent of each other. In particular, it was shown by FISH that the viral PB1 and PA segments of Influenza A H1N1 virus (A/WSN/1933) display a high degree of co-localization within the cytoplasm, suggesting that they co-occur in the same vRNP sub-bundle during ‘genome bundling’ [10]. As expected, mutations introduced into the genome packaging sequences of the A/WSN/1933 PA segment decreased its own incorporation into viral particles; however, and in sharp contrast to their potential association in vRNP sub-bundles, packaging of the presumably interacting viral PB1 segment was not affected [87]. Similarly, mutating the packaging sequences of the WSN PB1 segment diminished its own packaging but had no effect on the incorporation of the viral PA segment [87]. Considering those discrepancies it is unclear whether ‘genome bundling’ is indeed guided by genome packaging sequences (Figure 1a) or by yet to be identified alternative molecular determinants (Figure 1b). In order to solve this enigma at least in part, it will be essential to investigate vRNP sub-bundle composition by means of FISH, using aforementioned packaging sequence mutants to see whether mutations altering ‘genome packaging’ do also affect ‘genome bundling’.

In summary, the incorporation of a full genome set into progeny viral particles is a highly coordinated process exploiting fundamental cellular processes and sophisticated interplay between viral components. However, due to their inherent complexity, very little is known about ‘genome packaging’ and the role of ‘genome bundling’. Deciphering the molecular details of both processes might eventually allow the design of intervention strategies to deter IAV, which is the causative agent for annually high morbidity and mortality in the human population.
